# Bilirubin-Induced Oxidative Stress Leads to DNA Damage in the Cerebellum of Hyperbilirubinemic Neonatal Mice and Activates DNA Double-Strand Break Repair Pathways in Human Cells

**DOI:** 10.1155/2018/1801243

**Published:** 2018-11-26

**Authors:** Vipin Rawat, Giulia Bortolussi, Silvia Gazzin, Claudio Tiribelli, Andrés F. Muro

**Affiliations:** ^1^International Centre for Genetic Engineering and Biotechnology (ICGEB), Padriciano 99, 34149 Trieste, Italy; ^2^Centro Studi Fegato, Fondazione Italiana Fegato, Area Science Park, Campus Basovizza Trieste, Italy

## Abstract

Unconjugated bilirubin is considered a potent antioxidant when present at moderate levels. However, at high concentrations, it produces severe neurological damage and death associated with kernicterus due to oxidative stress and other mechanisms. While it is widely recognized that oxidative stress by different toxic insults results in severe damage to cellular macromolecules, especially to DNA, no data are available either on DNA damage in the brain triggered by hyperbilirubinemia during the neonatal period or on the activation of DNA repair mechanisms. Here, using a mouse model of neonatal hyperbilirubinemia, we demonstrated that DNA damage occurs *in vivo* in the cerebellum, the brain region most affected by bilirubin toxicity. We studied the mechanisms associated with potential toxic action of bilirubin on DNA in *in vitro* models, which showed significant increases in DNA damage when neuronal and nonneuronal cells were treated with 140 nM of free bilirubin (Bf), as determined by *γ*H2AX Western blot and immunofluorescence analyses. Cotreatment of cells with N-acetyl-cysteine, a potent oxidative-stress inhibitor, prevented DNA damage by bilirubin, supporting the concept that DNA damage was caused by bilirubin-induced oxidative stress. Bilirubin treatment also activated the main DNA repair pathways through homologous recombination (HR) and nonhomologous end joining (NHEJ), which may be adaptive responses to repair bilirubin-induced DNA damage. Since DNA damage may be another important factor contributing to neuronal death and bilirubin encephalopathy, these results contribute to the understanding of the mechanisms associated with bilirubin toxicity and may be of relevance in neonates affected with severe hyperbilirubinemia.

## 1. Introduction

Neonatal jaundice is a frequent condition resulting mainly from the temporary delay in the activation of bilirubin conjugation in the liver [1] or from genetic mutations in the UGT1A1 gene, with the permanent loss of bilirubin conjugation activity, a condition such as the Crigler-Najjar syndromes [2]. Prolonged and severe hyperbilirubinemia may be toxic for the developing nervous system, resulting in serious neurological damage and death associated with kernicterus [3].

During bilirubin-induced neurotoxicity, different molecular pathways are activated, ranging from mitochondrial damage and oxidative stress to endoplasmic reticulum (ER) stress response and inflammation [4]. Several *in vitro* and *in vivo* data demonstrated a main role of oxidative stress in cytotoxicity, when high, toxic, bilirubin concentrations are present. In fact, incubation of synaptic vesicles, tissue culture cells, and primary cell cultures of neurons, oligodendrocytes, and astrocytes with bilirubin resulted in increases in oxidative stress and cytotoxicity [4–12]. *In vivo* studies in Gunn rats show high levels of lipid peroxidation by sulphadimethoxine-induced hyperbilirubinemia [13]. Studies in hyperbilirubinemic Ugt1^−/−^ mice showed an impairment of antioxidant defenses with the activation of key oxidative stress markers [14–16].

It is widely recognized that increases in oxidative stress by a number of different insults result in severe damage to cellular macromolecules, especially to DNA [17, 18]. However, few data are available on the effects of bilirubin in DNA damage and its repair mechanisms.

Studies of DNA damage in peripheral lymphocytes of hyperbilirubinemic newborns do not provide conclusive evidence [19–24], while during postmortem analyses of cases affected by bilirubin encephalopathy, 8-hydroxy-2′-deoxyguanosine (8-OHG) was present only in half of the cases [25]. More recent data show increased DNA strand breaks and apurinic sites in peripheral blood mononuclear cells (PBMCs) of Gunn rats (a nonlethal model of neonatal hyperbilirubinemia), compared to wild-type (WT) Wistar controls [26], and the increases in 8-OHG and *γ*H2AX concentrations after treating tissue culture cells with bilirubin [12].

Still, no information is available regarding the effects of bilirubin on DNA damage *in vivo*, during neonatal development, the most critical period of neuronal susceptibility to bilirubin toxicity during postnatal brain development, and *in vitro* on DNA damage and DNA repair mechanisms in tissue culture cells treated with bilirubin.

In the present work, we studied the generation of DNA damage by bilirubin *in vivo*, using a very severe lethal mouse model of neonatal hyperbilirubinemia closely reproducing the human condition [27]. We also investigated the induction of DNA damage by bilirubin in neuronal and nonneuronal cells and the activation of the main DNA repair pathways: homologous recombination (HR) and nonhomologous end joining (NHEJ).

## 2. Materials and Methods

### 2.1. Animals

Mice were housed and handled according to institutional guidelines. Ethical and experimental procedures were reviewed and approved by the ICGEB board, with full respect to the EU Directive 2010/63/EU for animal experimentation. *Ugt1^−/−^* mice with a FVB/NJ background were generated as previously described [27, 28]. Homozygous mutant animals were obtained from heterozygous *Ugt1^+/−^* matings. *Ugt1^+/+^* (WT) littermates were used as controls. Animals used in this study were at least 99.8% FVB/NJ genetic background, obtained after more than ten backcrosses with WT FVB/NJ mice. Mice were kept in a temperature-controlled environment with a 12/12 h light/dark cycle. They received a standard chow diet and water *ad libitum*.

### 2.2. Biochemical Analysis of Plasma Samples

**Figure 1:**
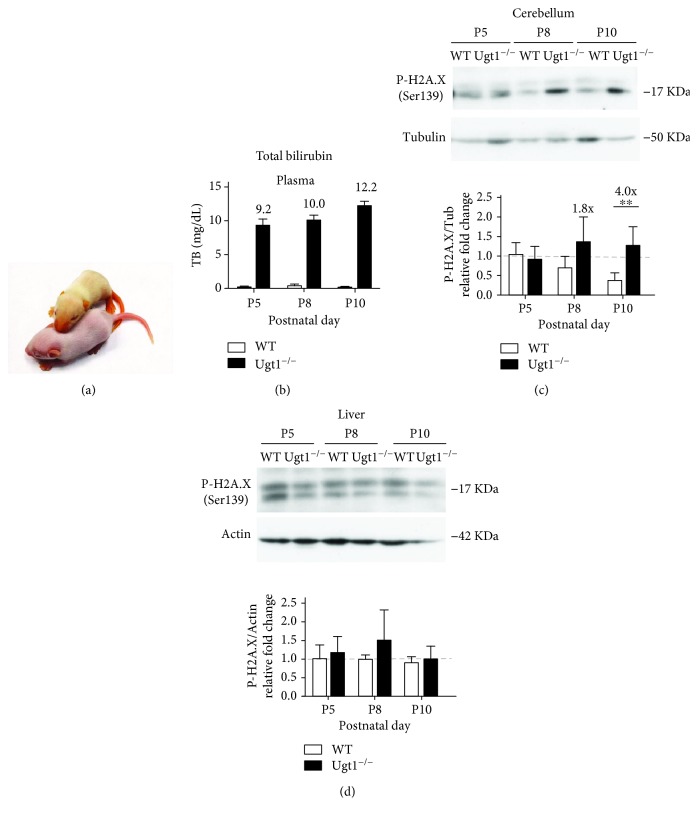
*In vivo* analysis of DNA damage in hyperbilirubinemic mice. Hyperbilirubinemic Ugt1^−/−^ mice (a) were used to determine the levels of plasma bilirubin (b) and *γ*H2AX (c and d) at different time points (P5, P8, and P10). Western blot analyses of the cerebella and liver of wild-type (WT) and mutant mice were performed using a *γ*H2AX-specific antibody. The *γ*H2AX signal was quantified and normalized (*β*-tubulin for the cerebellum, and actin for the liver). The values above the bars (c; P8 and P10) indicate the fold increase of the signal present in mutant tissues with respect to age-matched WT tissues. Five animals were analyzed per each genotype and time point. Two-way ANOVA; ^∗∗^ *P* < 0.01.

Total bilirubin (TB) determinations in plasma were performed as previously described [27] and in the same animals that were used in Figure 1 by Western blot (WB).

### 2.3. Preparation of Total Protein Extracts and Western Blot from Tissues

The selected organs were analyzed as previously described [28]. Briefly, cerebella and livers harvested at postnatal day (P) 5, P8, and P10 were dissected and homogenized in lysis buffer (150 mM NaCl, 1% NP-40, 0.5% DOC, 0.1% SDS, 50 mM Tris HCl pH 8, 2× protease inhibitors, 1× phosphatase inhibitors) and analyzed by WB. Primary anti-*γ*H2AX (Ser 139) antibody (Millipore, Milan, Italy) was diluted 1 : 2000 in 5% milk Tris HCl-buffer saline-Tween (TBST); anti-PARP antibody (#9542, Cell Signaling) was diluted 1 : 5000 in 5% milk TBST. Anti-tubulin mAb E7 (Developmental Studies Hybridoma Bank, Iowa City, IA) or anti-actin (Sigma-Aldrich) antibodies were used as loading controls.

### 2.4. Cell Culture

SH-SY5Y cells were cultured in Eagle's minimum essential medium F12 (EMEM/F12, Sigma-Aldrich) supplemented with 15% fetal calf serum (FCS), 1% antibiotic + antimycotic solution (Sigma-Aldrich A5955), 1% MEM nonessential amino acid solution (Sigma-Aldrich M7145). HeLa DR-GFP and HeLa cells were cultured in Dulbecco's modified Eagle's medium (DMEM, Thermo Fisher Scientific, Gibco) supplemented with 10% FCS and 1% antibiotic + antimycotic solution (Sigma-Aldrich A5955). The HeLa DR-GFP cell line was generously provided by Dr. Enrico Avvedimento [29].

### 2.5. Unconjugated Bilirubin (UCB) Treatment

UCB was purified using the method of Ostrow et al. [30], divided in aliquots and stored at −20°C until use. The total UCB concentration needed to reach the concentration of free bilirubin (Bf) 70 and 140 nM was determined using a previously described protocol [31]. UCB needed to reach the desired concentrations of Bf dissolved in 0.6% DMSO, and appropriate amounts of UCB were added to culture media in order to reach the desired Bf and measured spectrophotometrically at 468 nm.

### 2.6. MTT Assay for Cellular Viability

The MTT test for cellular viability was performed as previously described [32]. For a detailed description, see Supplementary Materials (available here).

### 2.7. Cell Transfection

For a detailed description, see Supplementary Materials.

### 2.8. SDS PAGE and WB Analysis

For a detailed description, see Supplementary Materials.

### 2.9. Immunofluorescence (IF) Analysis

For a detailed description, see Supplementary Materials.

### 2.10. Statistical Analyses

The Graphpad Prism package was used to analyze the data. All data were represented as mean ± SD. *P* < 0.05 was considered significant. Depending on the experimental design, Student's *t*-test, one-way ANOVA, or two-way ANOVA with Bonferroni's post hoc comparison tests was used, as indicated in the legends to the figures.

## 3. Results

### 3.1. Neonate Mice with Severe Hyperbilirubinemia Have DNA Damage in the Cerebellum

We have previously demonstrated key roles of oxidative stress and inflammation in bilirubin neurotoxicity in the cerebellum of hyperbilirubinemic Ugt1^−/−^ neonatal mice [15, 16]. In the present study, we investigated whether bilirubin-induced oxidative stress is associated with increased DNA damage *in vivo* in hyperbilirubinemic Ugt1^−/−^ neonatal mice (Figure 1(a)). In these animals, plasma bilirubin levels increased during the first days of life due to the absence of glucuronidation activity (Figure 1(b)), reaching toxic levels that result in the death of all pups before P15 (with 50% mortality at P11) [16, 27]. Since the cerebellum is the most affected region of the brain in this mouse strain, with no other organs damaged [27, 28, 33], we determined the levels of the phosphorylated histone H2AX (*γ*H2AX) in the cerebellum of mutant pups at different time points. *γ*H2AX is a well-recognized, highly specific, and sensitive molecular marker for monitoring DNA damage [34]. In addition, we also analyzed liver samples of the same animals to verify whether other organs were affected by bilirubin-induced DNA damage. WB of cerebellar *γ*H2AX showed a time-dependent increase in the levels of *γ*H2AX in the cerebellum of Ugt1^−/−^ mice versus age-matched normobilirubinemic WT littermates, with significant differences at P8 and P10 (Figure 1(c)), time points that are critical for neuro-susceptibility to bilirubin toxicity [27]. On the contrary, no obvious differences were observed in the liver (Figure 1(d)). These results demonstrated that bilirubin-induced DNA damage occurred during severe neonatal hyperbilirubinemia, suggesting that DNA damage may be associated with bilirubin neurotoxicity *in vivo*.

### 3.2. Bilirubin-Induced DNA Damage In Vitro

After having demonstrated the presence of DNA damage *in vivo* in the cerebellum of hyperbilirubinemic Ugt1^−/−^ mice, we used a simpler experimental setup using *in vitro* cell cultures to study the associated mechanisms.

We first confirmed the toxicity of bilirubin to neuronal SH-SY5Y human cells by incubating the cells with the quantified Bf concentrations in the presence of FCS. Bilirubin concentration was adjusted to reach 70 or 140 nM of Bf using the modified peroxidase assay [31] as described in Materials and Methods. Cell viability decreased after 4 h treatment with both 70 and 140 nM Bf concentrations and further decreased at 24 h, reaching 60% with 140 nM Bf (Supplementary Figure 1). Based on these findings and previous results [12, 32, 35–37], the 140 nM Bf concentration was selected for the next set of experiments.

**Figure 2:**
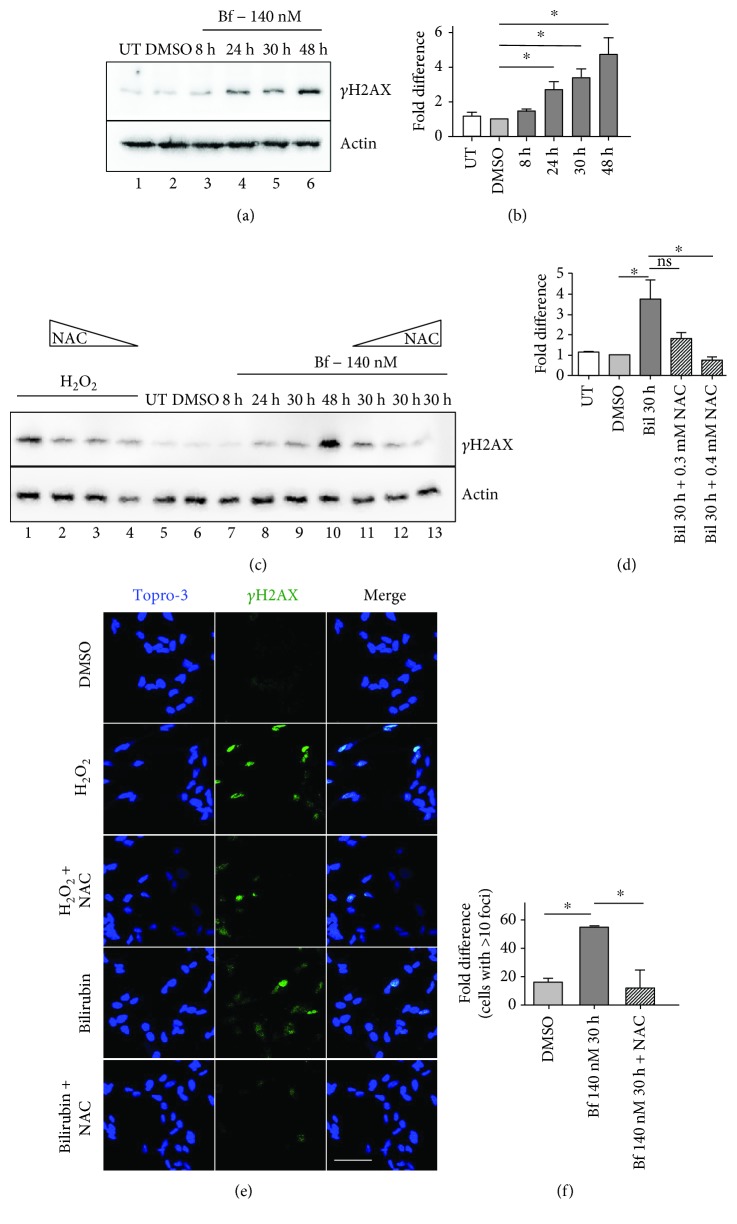
Bilirubin-induced oxidative stress causes DNA damage in SH-SY5Y cells. SH-SY5Y cells were treated with 140 nM free bilirubin (Bf) for different time points (8, 24, 30, and 48 h; lanes 3-6). DMSO (0.6% DMSO, bilirubin solvent) served as controls (lane 2). Cells were collected at the indicated time points, and the levels of *γ*H2AX were determined by Western blot analysis and quantified (mean of three independent experiments (b)); (c, d) SH-SY5Y cells were treated with H_2_O_2_ (lanes 1-4) or 140 nM Bf for different times, as indicated (lanes 7-13). In lanes 2-4 and 11-13, cells were treated with H_2_O_2_ or 140 nM Bf for 48 h plus N-acetyl-cysteine (NAC) treatment (0.1, 0.3, or 0.4 mM). *γ*H2AX was determined by Western blot, quantified and normalized (actin). (d) Quantification of two independent experiments; (e, f) SH-SY5Y cells were treated with H_2_O_2_ or 140 nM bilirubin for 30 h, with or without NAC (0.4 mM) treatment, as described in Materials and Methods. *γ*H2AX foci were determined by immunofluorescence. Cells containing more than 10 foci were considered positive. The scale bar corresponds to 30 *μ*M; (f) quantification from two independent experiments. Values represented as mean ± SD. One-way ANOVA with Bonferroni's post hoc test was applied for statistical analysis. ^∗^ *P* < 0.05; NS: not significative; UT: untreated cells.

To determine the toxic effects of high levels of bilirubin on DNA, cells were incubated with 140 nM Bf for different times (up to 48 h) and the levels of *γ*H2AX determined by WB analysis. Incubation of SH-SY5Y cells with bilirubin resulted in a significant increase in the *γ*H2AX signal, reaching a maximum at 48 h (Figures 2(a) and 2(b)), confirming the presence of bilirubin-induced DNA damage as observed *in vivo* (Figure 1). Untreated (UT) and DMSO-treated (vehicle) cells showed similar *γ*H2AX levels, thereby ruling out any effect due to DMSO.

Next, to determine whether the observed increase in *γ*H2Ax was the consequence of bilirubin-induced oxidative stress, cells were incubated with bilirubin in the presence or absence of N-acetyl cysteine (NAC), a powerful exogenous antioxidant. Incubation of cells with H_2_O_2_, a potent generator of reactive oxygen species (ROS), was used as a positive control. In fact, the levels of *γ*H2AX induced by H_2_O_2_ decreased after NAC treatment (Figure 2(c), lanes 2-4). As observed in lanes 11-13 (Figures 2(c) and 2(d)), NAC treatment inhibited DNA damage in a dose-dependent manner.

We further confirmed these results by immunofluorescence (IF) analysis of treated cells (Figures 2(e) and 2(f)). Treatment of SH-SY5Y cells with bilirubin resulted in ~50% of cells having *γ*H2AX foci, proportion that was reduced to control levels with NAC treatment.

These results indicate that bilirubin-induced oxidative stress appears to be the main mechanism causing DNA damage *in vitro*.

To gain a deeper insight in the associated mechanisms, we determined poly(ADP-ribose) polymerase (PARP) levels in the cerebella of mutant mice at P10. PARP has an important role in DNA repair, and at the same time, it modulates inflammation by activating nuclear factor *κ*B (NF*κ*B) [38]. We observed an increase in the cleaved form of PARP at P10 (Supplementary Figure 2), concomitantly with the maximum increase in inflammatory response reported for this animal model [16]. These data suggest that bilirubin-induced DNA damage response and inflammation may be associated *in vivo*.

### 3.3. Bilirubin Stimulates Nonhomologous End Joining (NHEJ) and Homologous Recombination (HR) DNA Repair Pathways *In Vitro*

We then investigated the mechanisms of DNA double-strand break (DSB) repair in the presence of toxic levels of Bf. To determine the effect of bilirubin in NHEJ and HR DNA repair pathways, we used HeLa cells and the HeLa DR-GFP cell lines [29]. We first determined HeLa DR-GFP cell viability after a bilirubin toxic insult at different time points by exposing cells to 140 nM Bf (Supplementary Figure 3). As expected, the viability of cells decreased in a time-dependent manner, reaching 45% after 24 h.

**Figure 3:**
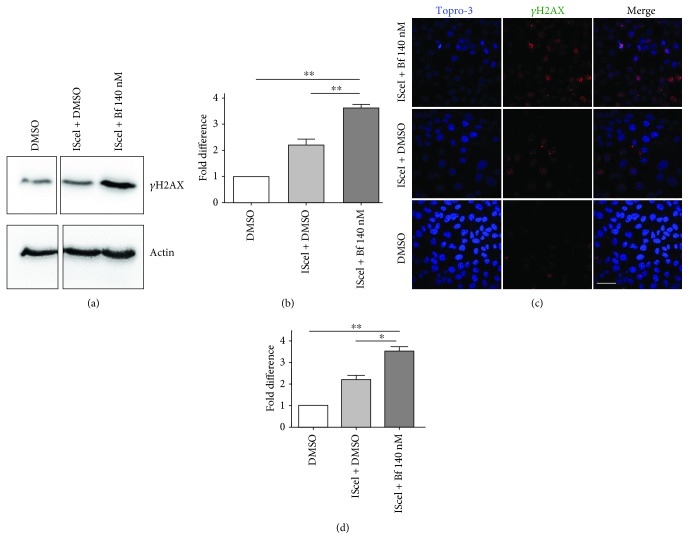
Bilirubin-induced DNA damage in HeLa DR-GFP cells. (a, b) Western blot analysis of HeLa DR-GFP cells transfected with a plasmid encoding the ISceI endonuclease (pCBA IsceI) and treated with DMSO (control) or 140 nM free bilirubin (Bf). *γ*H2AX levels were determined by Western blot analysis, quantified and normalized (actin). (b) Quantification from three independent experiments. (c, d) HeLa DR-GFP cells were treated as described in (a) and (b), and *γ*H2AX foci were determined by immunofluorescence. Cells containing more than 10 foci were considered positive. (f) Quantification from two independent experiments. One-way ANOVA with Bonferroni's *post hoc* test was used to perform statistical analysis. ^∗^ *P* < 0.05; ^∗∗^ *P* < 0.01.

To study the HR-mediated DNA repair mechanism *in vitro*, we used the HeLa DR-GFP cell line [29]. We determined the levels of *γ*H2AX by WB and IF in these cells upon bilirubin and ISceI treatment combinations. We observed that transfection with the ISceI-encoding plasmid increased *γ*H2AX signaling, as determined both by WB and IF. *γ*H2AX levels were further increased in ISceI-transfected cells treated with 140 nM Bf (Figure 3). These results indicated that the amount of DSB is increased by bilirubin treatment.

**Figure 4:**
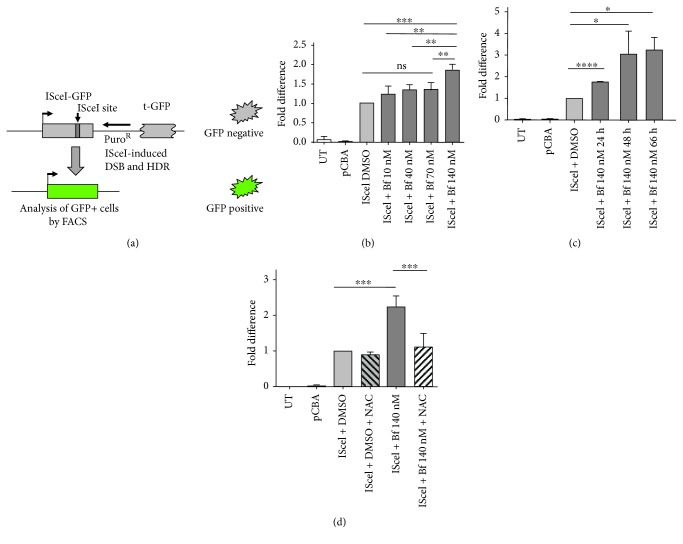
Bilirubin-induced oxidative stress modulates homologous recombination. (a) Schematic diagram of the DR-GFP system. (b) HeLa DR-GFP cells were transfected with a plasmid encoding the IsceI endonuclease (pCBA IsceI) and treated with different amounts of Bf (from 10 to 140 nM). Cells were analyzed for GFP fluorescence by FACS analysis 72 h posttransfection. Data were normalized for transfection efficiency using renilla luciferase activity, encoded by a cotransfected plasmid. (c) HeLa DR-GFP cells were transfected with the ISceI plasmid and treated for different times with 140 nM Bf. Data was normalized for transfection efficiency by transfecting cells with eGFP in parallel wells. (d) HeLa DR-GFP cells were cotransfected with a plasmid encoding IsceI (pCBA IsceI) and plasmid encoding renilla luciferase (pHRG-TK renilla). 6-7 h after transfection, 140 nM Bf was added to cells, with or without N-acetyl cysteine (NAC) treatment, as described in Materials and Methods. Data were normalized for transfection efficiency using renilla activity in the same well. Values were represented as mean ± SD. One-way ANOVA with Bonferroni's *post hoc* test was used to perform statistical analysis. ^∗^ *P* < 0.05; ^∗∗^ *P* < 0.01; ^∗∗∗^ *P* < 0.001; ^∗∗∗∗^ *P* < 0.0001.

The HeLa DR-GFP HR reporter cell line contains a single genomic integration of the DR-GFP recombination reporter construct [29], which has two differentially inactivated versions of a tandemly repeated (DR) GFP, developed by Pierce et al. [39]. The upstream GFP gene (Cassette I), under the transcriptional control of the CMV enhancer and chicken *β*-actin promoter, is interrupted by an ISceI restriction enzyme site, a rare-cutting endonuclease [40]. Introduction of the ISceI site leads to the formation of in-frame stop codons inactivating Cassette I. The downstream GFP fragment (Cassette II) is truncated at the 5′ and 3′ ends, resulting in a truncated and inactive GFP product (Figure 4(a)). Transient transfection with a plasmid encoding the ISceI endonuclease generates a DSB, which enhances HR between the two cassettes and thereby production of active GFP that can be quantified by flow cytometry (Figure 4(a)) [29].

We then determined the modulation of HR-mediated DNA repair by bilirubin-induced oxidative stress using the HeLa DR-GFP cell line (Figure 4(a)) [29]. Transfection of DR-GFP cells with the IsceI-encoding plasmid resulted in a significant increase in GFP-positive cells compared to the pCBA control plasmid (Figure 4(b) and Supplementary Figure 4). Incubation of the ISceI-transfected cells with increasing amounts of Bf resulted in a dose-dependent increase of HR, reaching a statistically significant difference at 140 nM Bf. Similarly, we also observed a time-dependent increase in HR in cells treated with ISceI plus bilirubin, reaching a plateau at 66 h (Figure 4(c) and Supplementary Figure 4). To determine whether the increase in HR by bilirubin was associated with oxidative stress, ISceI-transfected cells were treated with bilirubin and NAC. We observed that NAC treatment reduced HR to the levels of the IsceI control (without bilirubin treatment) (Figure 4(d) and Supplementary Figure 4) suggesting that the increase in HR was associated with the bilirubin-induced increase in oxidative stress.

**Figure 5:**
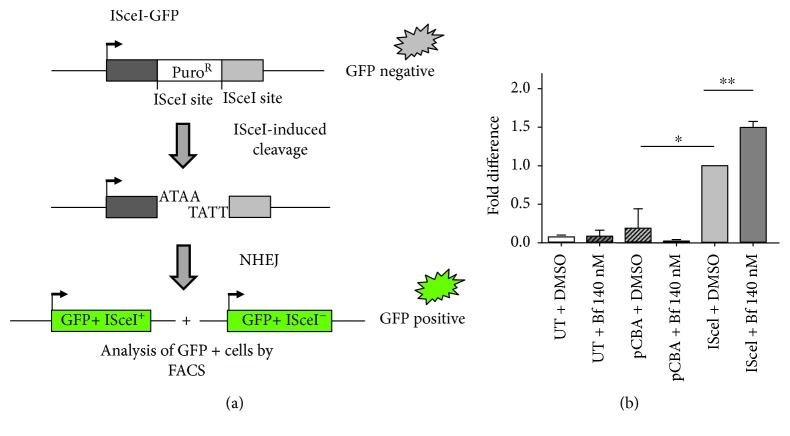
Bilirubin-induced NHEJ in HeLa cells. (a) Schematic representation of pIM EJ5 GFP reporter plasmid used for nonhomologous end joining (NHEJ) experiments. (b) HeLa cells were cotransfected with pIM EJ5 GFP plasmid encoding the NHEJ reporter and a plasmid encoding renilla luciferase (pHRG-TK renilla). Bilirubin (140 nM Bf) or DMSO (control) was added to cells 6-7 h after transfection. Cells were analyzed for GFP fluorescence 72 h posttransfection by fluorescence-activated cell sorting (FACS) analysis. The graph represents the quantification of two independent experiments. Data were normalized for transfection efficiency using renilla luciferase activity. One-way ANOVA with Bonferroni's *post hoc* test was used to perform statistical analysis. Values are represented as mean ± SD. ^∗^ *P* < 0.05; ^∗∗^ *P* < 0.01.

Next, to determine the potential increase in NHEJ, we used a reporter vector specifically designed to detect multiple classes of NHEJ events [41]. The EJ5-GFP vector contains a promoter that is separated from a GFP-coding cassette by a puromycin-resistance gene. This gene is flanked by two ISceI sites which is a rare-cutting endonuclease [40]. Upon digestion with ISceI provided by a second plasmid, the puromycin cassette is removed and the two ISceI-induced DSBs are joined by NHEJ. This results in the restoration of the GFP gene transcription by the promoter, now joined to the GFP expression cassette. GFP-positive cells were quantified by FACS analysis (Figure 5(a) and Supplementary Figure 5).

Hela cells were transfected with the EJ5-GFP reporter plasmid, with or without the ISceI-coding plasmid (or pCBA control plasmid), and treated with 140 nM Bf or DMSO (vehicle). A plasmid encoding renilla luciferase (pHRG-TK renilla) was used to normalize for differences in transfection efficiency. Incubation with ISceI plus DMSO resulted in a significant increase in GFP-positive cells. The proportion of GFP-positive cells further increased after the incubation of the cells with 140 nM Bf, strongly suggesting the modulation of NHEJ by bilirubin.

## 4. Discussion

Bilirubin is considered a physiologically important antioxidant [42], with beneficial effects at mildly elevated concentrations, and can neutralize ROS and prevent oxidative damage [43–53]. However, at higher concentrations, bilirubin is toxic to the developing brain, leading to bilirubin encephalopathy or, in the most severe cases, to irreversible neurological damage and death due to kernicterus [3, 54]. Several *in vitro* and *in vivo* studies, including ours, have shown that oxidative stress generated by elevated concentrations of bilirubin is a key mechanism of bilirubin-induced cell toxicity [5, 8–15, 32].

In the present study, we have shown the effects of toxic concentrations of bilirubin to induce DNA damage and modulate DNA repair mechanisms. We first studied the levels of the sensitive DNA damage marker *γ*H2AX [34] in the cerebella of Ugt1^−/−^ mice versus age-matched normobilirubinemic littermates, revealing the presence of DNA damage *in vivo*. Notably, since these animals reproduce the major features of the Crigler-Najjar syndrome type I, such as the absence of bilirubin glucuronidation activity, severe neonatal hyperbilirubinemia, bilirubin neurotoxicity with cerebellar abnormalities, and death soon after birth if untreated [27, 28], these results may be clinically relevant.

DNA lesions may result in chromosomal translocation, deletions, and cell cycle arrest by stalling of replication forks [55]. Accordingly, alterations in cell proliferation by bilirubin have already been proposed [56–59] and have been documented in both tissue culture cells exposed to bilirubin [12] and the cerebellum of Gunn rats [60]. Likewise, the apoptosis-mediated neuronal death observed in the cerebellum of Ugt1^−/−^ pups [15, 16, 27] could be associated to some extent with the increased oxidative stress-induced DNA lesions in these animals.

DNA damage by bilirubin in Crigler-Najjar patients has never been directly addressed. Studies in babies with neonatal hyperbilirubinemia demonstrated DNA damage effects on peripheral blood after intensive phototherapy treatment. However, these studies showed controversial results regarding the presence of DNA damage in untreated (or prephototherapy) jaundiced babies compared to control normal groups [19–22, 24] and were performed in peripheral cells, which may have different susceptibility to bilirubin-induced oxidative stress than bilirubin-target organs. In fact, we have shown a time-dependent increase in DNA damage in the cerebellum, which is the most affected organ in the Ugt1^−/−^ hyperbilirubinemic model [27], while no DNA damage occurred in an unaffected tissue such the liver, which presents a higher total tissue bilirubin concentration compared to the cerebellum [61]. This observation is very intriguing as previous studies suggested the induction of oxidative stress in liver cell lines after bilirubin treatment [9, 37], an effect not observed *in vivo*. These discrepancies may be related to differences between *in vitro* and *in vivo* conditions, to the higher susceptibility of the developing cerebellum to oxidative stress (compared to the liver), and to differences in the levels of antioxidant enzymes in the liver vs. cerebellum [62].

Importantly, when we investigated the effects of bilirubin *in vitro* by incubating neuronal and nonneuronal cells with concentrations of bilirubin normally found in jaundiced patients, we not only reported the induction of DNA damage as determined by *γ*H2AX analysis but also demonstrated that it is caused by the prooxidant toxic action of the pigment. In fact, treatment with NAC, a potent antioxidant, prevented bilirubin-induced DNA damage and also DNA damage generated by H_2_O_2_, a widely recognized ROS generator and DNA damage inducer. These results strongly support the idea that bilirubin-induced oxidative stress is an important determinant of neurotoxicity [7, 37] and that DNA damage is caused by the increase in ROS upon bilirubin exposure. We have also reported the activation of PARP, suggesting a close connection between inflammation and DNA damage by bilirubin-induced toxicity. Interestingly, PARP modulates inflammation through the activation of NF*κ*B [38], a transcription factor upregulated in this animal model [16] and in neuronal cell cultures exposed to toxic levels of bilirubin [36]. However, further studies will be necessary to provide deeper mechanistic insights.

Our data are in line with our previous published results, showing the increase in ROS levels after incubation of cells with bilirubin [11, 12]. Cells treated with bilirubin showed an increase in intracellular ROS associated with decreased survival [11]. SH-SY5Y cells treated with bilirubin differentially expressed proteins involved in oxidative stress response such as PARK7/DJ-1, a multifunctional neuroprotective protein, a key protective factor in UCB-induced cell damage [12]. Bilirubin treatment also resulted in the reduction of cell proliferation and increase of DNA damage, as observed by increases in 8-OHG and *γ*H2AX content [12]. Studies in hyperbilirubinemic Ugt1^−/−^ mice showed an increase in heme oxygenase-I (HO-1) levels in activated microglia cells in the cerebella at P8 and P10. However, other genes involved in oxidative stress response were unaffected [16], suggesting that the presence of a partial *in vivo* response to this severe insult is unable to prevent or revert the damage. When the cerebellar proteome of P4 Ugt1^−/−^ newborn mice bearing the more severe C57Bl/6 genetic background was analyzed, we observed the impairment of oxidative stress defenses and an increase in cell death in the cerebella of these mice [15].

The findings presented here raise important concerns on the potential risks of carcinogenesis and mutagenic consequences of bilirubin, when present at very high plasma concentrations. Although no significant DNA damage is found in Gilbert patients and Gunn rats [26], no information is available from patients having permanently higher levels of UCB such as Crigler-Najjar syndrome (CNS) type I and II patients. In addition, depending on the country heath system, these patients may undergo liver transplantation early in childhood, limiting the possibility to obtain information related to tumor development in high bilirubin conditions in the long term. The potential risks of carcinogenesis are also limited by other confounding factors present after liver transplantation, such as life-long immunosuppression to avoid organ rejection, a treatment that increases the risk of developing tumors [63, 64]. Thus, these results may be important for CNS patients that do not undergo liver transplantation early in age. Because phototherapy treatment is less efficacious after growth and pubertal development, adult CNS patients may have very high levels of plasma bilirubin with sporadic spikes of even higher levels of plasma bilirubin [65], putting them at risk of DNA damage. In fact, the accumulation of DNA lesions in other scenarios contributes to neuronal cell death [66], a phenomenon observed in specific regions of the brain both in hyperbilirubinemic patients and rodent animal models [15, 27, 67, 68].

The data presented in this work show that bilirubin increases DNA repair capacity as a result of the oxidative stress-induced DNA damage. Cells activate DNA damage response pathways to correct the genetic lesions. For DSB repair, two main mechanisms are active: NHEJ [69] and HR [70]. We evaluated HR and NHEJ DNA repair mechanisms using specific reporter systems [29, 39–41]. Both DNA repair mechanisms were stimulated by the bilirubin treatment, as demonstrated by a significantly increased frequency of GFP-positive cells after their incubation with bilirubin. The effect was also consequent to bilirubin-induced oxidative stress since NAC treatment reduced HR to basal levels. We propose that the stimulation of HR and NHEJ pathways may be an adaptive response of the cell to repair DNA damage mediated by bilirubin.

## 5. Conclusions

In spite of the many proposed mechanisms leading to neurotoxicity during neonatal severe hyperbilirubinemia, including oxidative stress, no data are available on DNA damage *in vivo* and in the response mechanisms triggered by hyperbilirubinemia.

The results presented here contribute to the understanding of the mechanisms associated with bilirubin neurotoxicity. We demonstrated that (a) DNA damage occurs *in vivo* in jaundiced newborn mice; (b) DNA damage is caused by bilirubin-induced oxidative stress; and (c) bilirubin treatment activates HR and NHEJ DNA repair mechanisms. Further studies are needed to reveal whether bilirubin-induced oxidative stress also results in RNA oxidation or affects the cell response at different levels.

We believe that these results are of relevance for neonates with severe hyperbilirubinemia and for patients suffering from the Crigler-Najjar syndrome, since DNA damage may contribute to neuronal death and bilirubin encephalopathy.

## Data Availability

The data used to support the findings of this study are included within the article and the Supplementary Materials.

## Supplementary Materials

Supplementary Materials Supplementary Figure 1: bilirubin is toxic to SH-SY5Y cells. Supplementary Figure 2: cerebella from mutant mice show PARP activation. Supplementary Figure 3: bilirubin treatment affects the viability of HeLa cells. Supplementary Figure 4: data from Figure 4. FACS analysis data of homologous recombination experiments. Supplementary Figure 5: data from Figure 5. FACS analysis data of nonhomologous end joining experiments.
