# Supplementary figures and images for: Bilirubin-Induced Oxidative Stress Leads to DNA Damage in the Cerebellum of Hyperbilirubinemic Neonatal Mice and Activates DNA Double-Strand Break Repair Pathways in Human Cells
Source: Oxid Med Cell Longev. 2018 Nov 26;2018:1801243. doi: 10.1155/2018/1801243 (PMC6287157; doi:10.1155/2018/1801243)

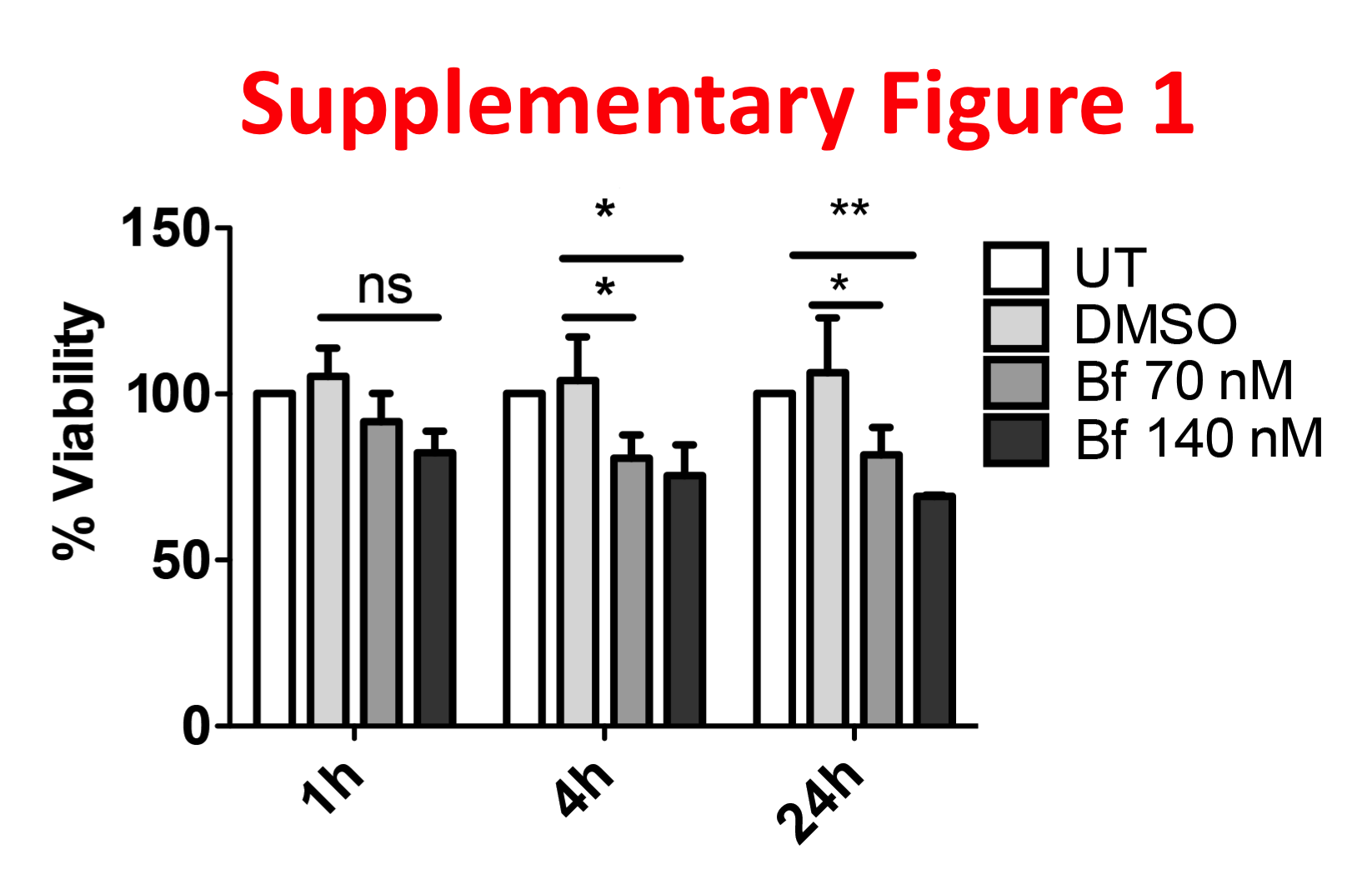

Supplement: Supplementary Materials — Supplementary Figure 1: bilirubin is toxic to SH-SY5Y cells. Supplementary Figure 2: cerebella from mutant mice show PARP activation. Supplementary Figure 3: bilirubin treatment affects the viability of HeLa cells. Supplementary Figure 4: data from Figure 4. FACS analysis data of homologous recombination experiments. Supplementary Figure 5: data from Figure 5. FACS analysis data of nonhomologous end joining experiments. [file 1801243.f1.zip › Supplementary Figure 1.tiff]

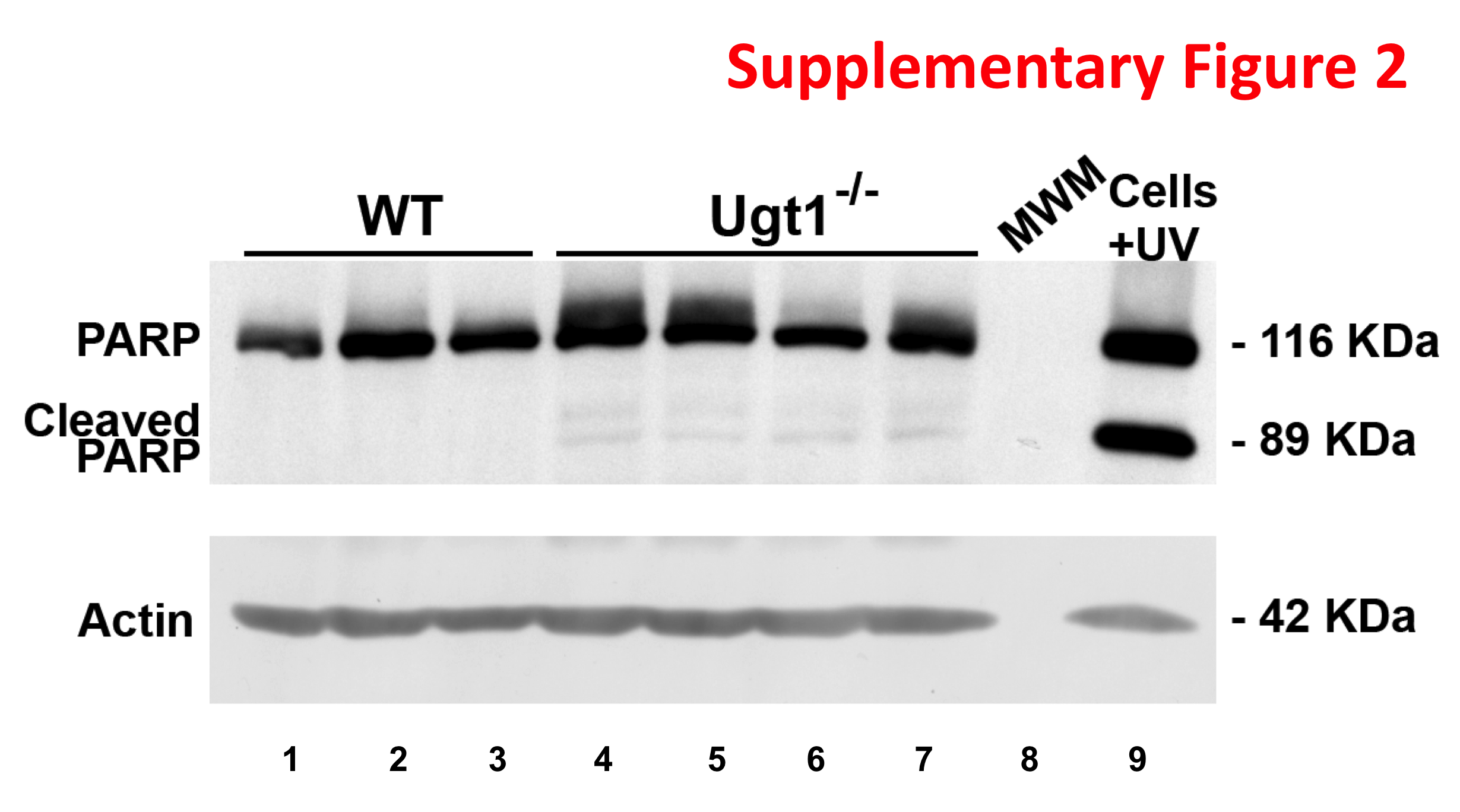

Supplement: Supplementary Materials — Supplementary Figure 1: bilirubin is toxic to SH-SY5Y cells. Supplementary Figure 2: cerebella from mutant mice show PARP activation. Supplementary Figure 3: bilirubin treatment affects the viability of HeLa cells. Supplementary Figure 4: data from Figure 4. FACS analysis data of homologous recombination experiments. Supplementary Figure 5: data from Figure 5. FACS analysis data of nonhomologous end joining experiments. [file 1801243.f1.zip › Supplementary Figure 2.tiff]

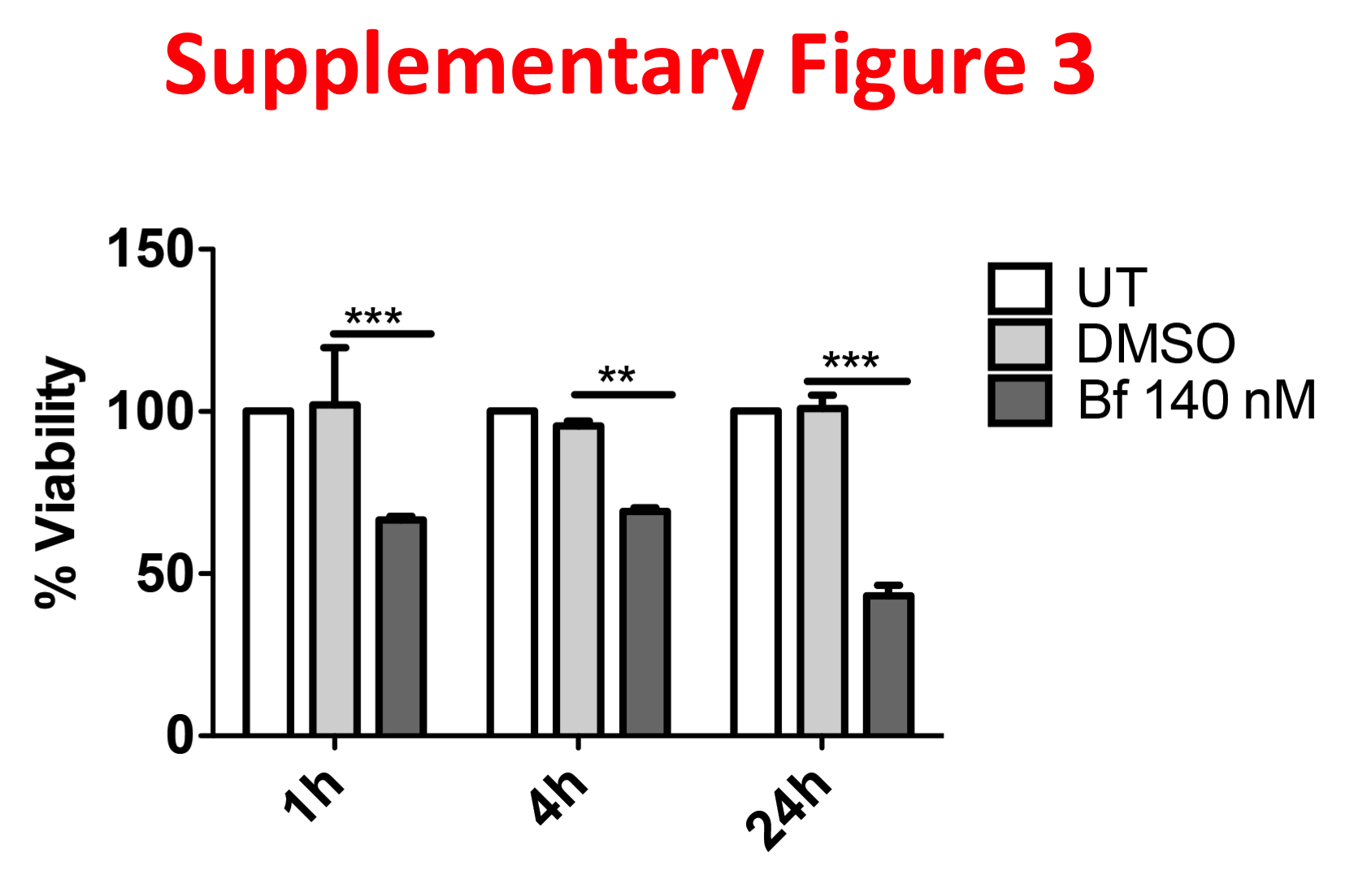

Supplement: Supplementary Materials — Supplementary Figure 1: bilirubin is toxic to SH-SY5Y cells. Supplementary Figure 2: cerebella from mutant mice show PARP activation. Supplementary Figure 3: bilirubin treatment affects the viability of HeLa cells. Supplementary Figure 4: data from Figure 4. FACS analysis data of homologous recombination experiments. Supplementary Figure 5: data from Figure 5. FACS analysis data of nonhomologous end joining experiments. [file 1801243.f1.zip › Supplementary Figure 3.tiff]

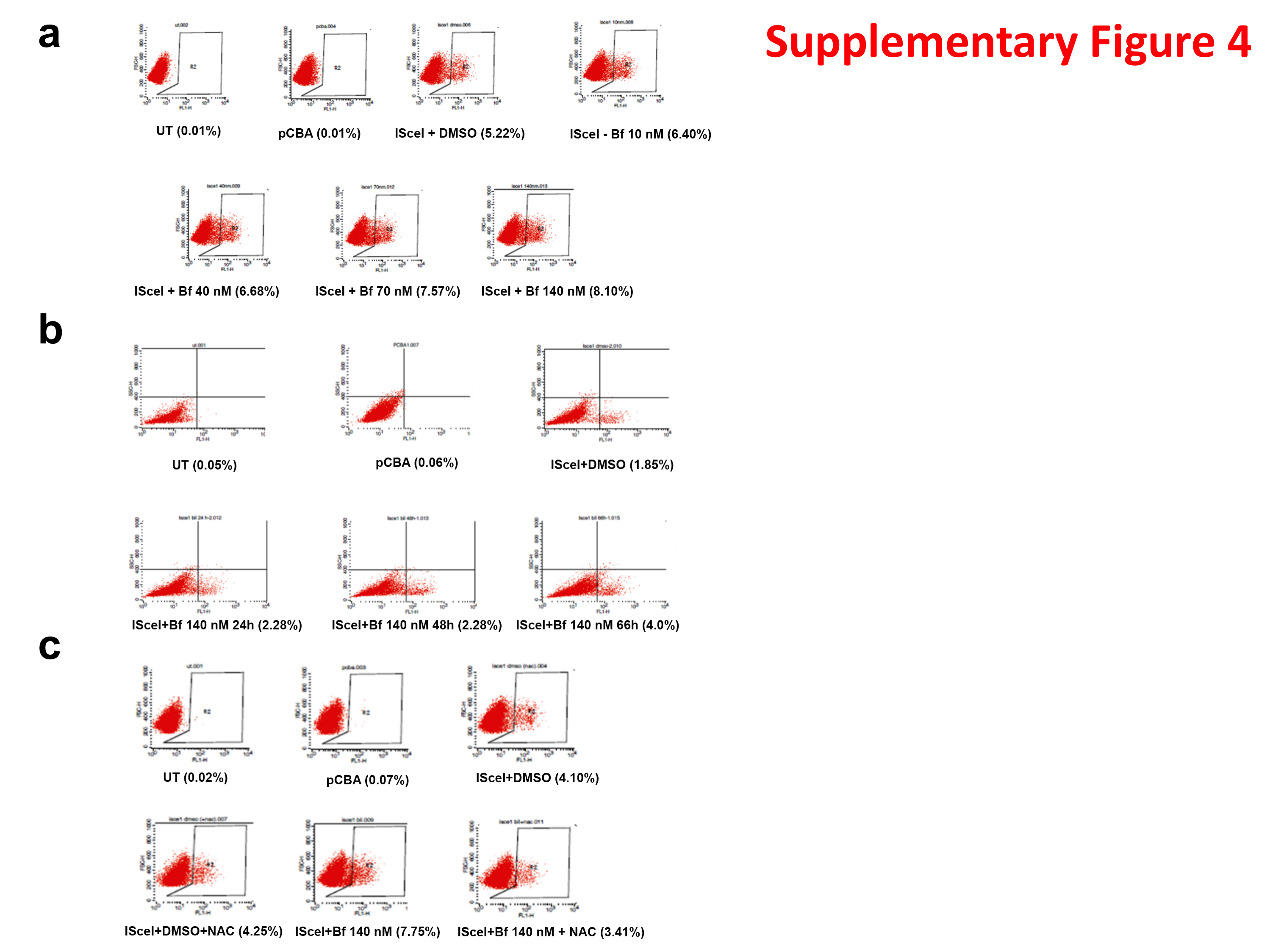

Supplement: Supplementary Materials — Supplementary Figure 1: bilirubin is toxic to SH-SY5Y cells. Supplementary Figure 2: cerebella from mutant mice show PARP activation. Supplementary Figure 3: bilirubin treatment affects the viability of HeLa cells. Supplementary Figure 4: data from Figure 4. FACS analysis data of homologous recombination experiments. Supplementary Figure 5: data from Figure 5. FACS analysis data of nonhomologous end joining experiments. [file 1801243.f1.zip › Supplementary Figure 4.tiff]

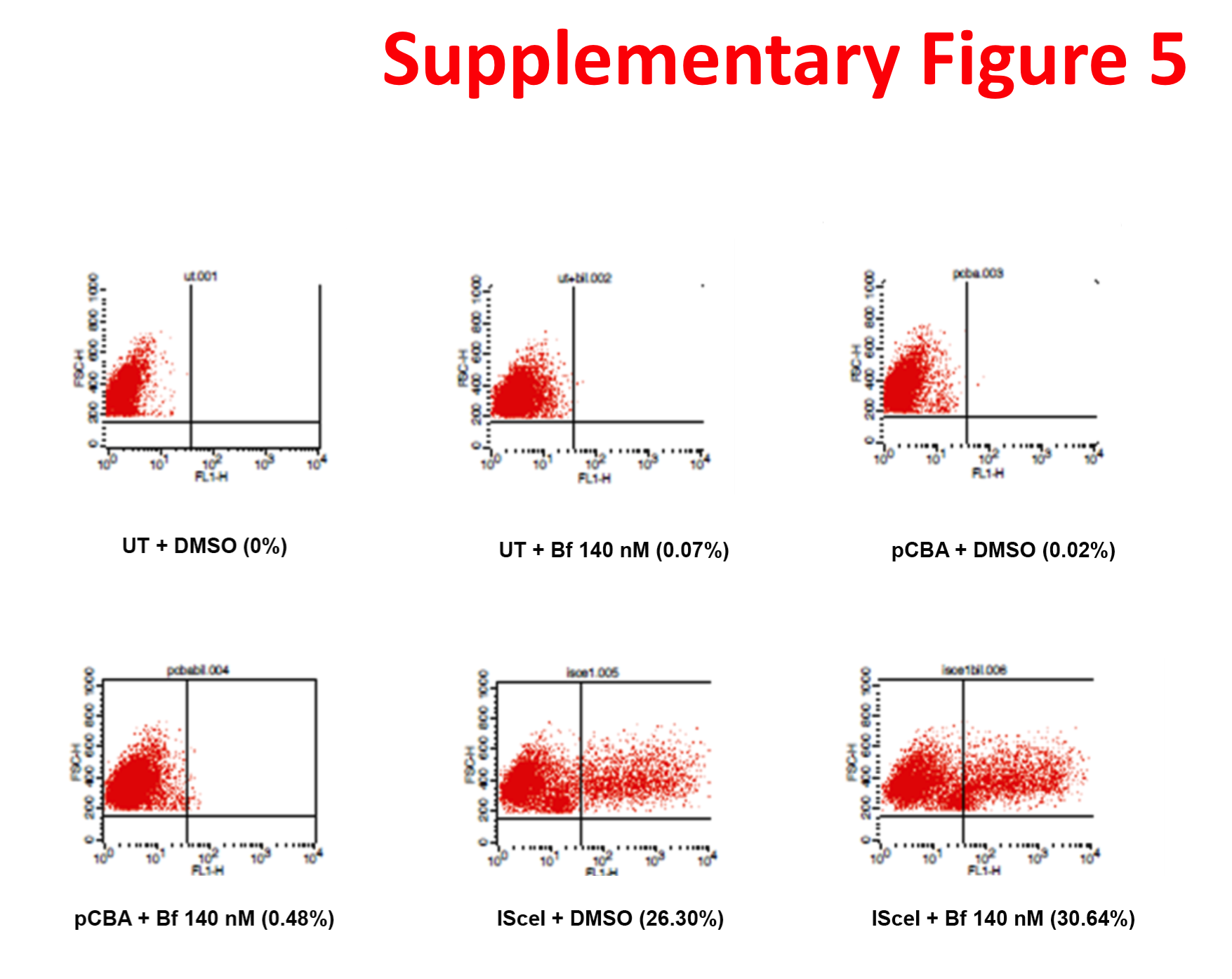

Supplement: Supplementary Materials — Supplementary Figure 1: bilirubin is toxic to SH-SY5Y cells. Supplementary Figure 2: cerebella from mutant mice show PARP activation. Supplementary Figure 3: bilirubin treatment affects the viability of HeLa cells. Supplementary Figure 4: data from Figure 4. FACS analysis data of homologous recombination experiments. Supplementary Figure 5: data from Figure 5. FACS analysis data of nonhomologous end joining experiments. [file 1801243.f1.zip › Supplementary Figure 5.tiff]
